# Should Caregivers Attempt to Assist Falling Patients?

**DOI:** 10.1093/geroni/igaa057.765

**Published:** 2020-12-16

**Authors:** Tatjana Bulat, Yvonne Friedman, Blake Barrett, Jason Lind, Margeaux Chavez, Linda Cowan, Marie Martin, Alicia Shaw

**Affiliations:** 1 JAH VA Hospital, Patient Safety Center of Inquiry, Tampa, Florida, United States; 2 Tampa VA Hospital, Tampa, Florida, United States; 3 VA North Texas, Dallas, Texas, United States; 4 Milwaukee VA, Milwaukee, Wisconsin, United States

## Abstract

This quality improvement project seeks to provide guidance on whether caregivers should attempt to “assist” fallers, and if so, the safest way to minimize injury to themselves and the faller. Primary aims were to: 1) Identify common characteristics of documented assisted falls, 2) Identify cases where injuries to patients and/or staff occur, and 3) Provide guidance to the clinical field. Data sources for this project includes secondary databases of assisted falls events as well as primary data collection using computer simulation. Initial results for 2 VA quality tracking databases of assisted falls over a 9-year period are presented. Qualitative matrix analyses were conducted for both assisted falls datasets, which separately examined patient and employee injuries related to assisted falls. Two trained qualitative experts analyzed 195 fall narratives from the datasets to develop insights about the most common fall scenarios that result in injury. The most commonly reported assisted falls scenarios included 1) related to toileting, 2) while ambulating, and 3) while transferring from wheelchair. Findings of these analyses indicate current documentation does not capture the nuance of assisted falls. Additional variables such as 1) the direction of the fall; 2) the fall scenario; 3) how staff sought to assist; 4) staff injury description and 5) and other key variables (patient symptoms, environmental factors) that could improve fall documentation and understanding of assisted falls. Preliminary efforts are providing information for development of a computer simulation using a virtual environment to repeatedly test common fall scenarios and influences of caregiver assistance.

